# N-Acetyl-L-Cysteine Protects Airway Epithelial Cells during *Respiratory Syncytial Virus* Infection against Mucin Synthesis, Oxidative Stress, and Inflammatory Response and Inhibits HSPA6 Expression

**DOI:** 10.1155/2022/4846336

**Published:** 2022-08-21

**Authors:** Lei Chi, Yuxia Shan, Zhenze Cui

**Affiliations:** Respiratory Department, Dalian Women and Children's Medical Group, China

## Abstract

*Objective. Respiratory syncytial virus* (RSV) infection is an important cause of hospitalization of children worldwide, leading to significant morbidity and mortality. RSV infection leads to increasing inflammatory and apoptosis events in the airway epithelium through mechanisms involving ROS generation. The antioxidant N-acetyl-L-cysteine (NAC) has been shown to inhibit influenza virus replication and to reduce the secretion of inflammatory and apoptotic mediators during virus infection. The study aims to investigate the effects of NAC on human bronchial epithelial cells BEAS-2B and HSPA6 expression during RSV infection. *Methods.* CCK-8 assays were performed to evaluate cell survival. The production of proinflammatory factors, TNF-*α*, IL-6, IL-1*β*, IL-18, and MUC5AC was examined by quantitative real-time PCR and ELISA. Oxidative stress was determined by reactive oxygen species (ROS), superoxide dismutase (SOD), malondialdehyde (MDA), and glutathione (GSH)/glutathione disulfide (GSSG) ratio. Immunoblotting analysis of epidermal growth factor receptor (EGFR) and its phosphorylation was performed. The antiviral effect of NAC was assessed by determining viral titers using plaque assay. *Results.* RSV infection reduced cell survival, promoted the release of proinflammatory factors, increased the ROS production and MDA concentration, and diminished the SOD activity and GSH/GSSG ratio, all which were attenuated by NAC treatment. Accordingly, NAC treatment inhibited the activation of EGFR and MUC5AC in BEAS-2B cells with RSV infection. Furthermore, NAC administration resulted in a marked decrease in RSV-induced HSPA6 expression in BEAS-2B cells. Concomitantly, EPB treatment led to an evident inhibition of RSV fusion gene and viral replication in RSV-infected BEAS-2B cells. *Conclusion.* This work supports the use of NAC to exert antimucin synthesis, anti-inflammatory, antioxidant, and antiviral effects on airway epithelium during RSV infection.

## 1. Introduction

Common respiratory viruses, including influenza, respiratory syncytial virus (RSV), human metapneumovirus, parainfluenza, rhinovirus, and adenovirus, are the main causes of viral respiratory infection [1]. RSV is one of the most important pathogenic infections in childhood, resulting in significant incidence rate and mortality [2]. In China, RSV diagnostic testing results from the 2014-2018 surveillance of acute respiratory infections showed that the incidence rate of RSV infection among children aged less than 5 years during 2014-2018 was 17.3%, 89.1% of RSV-positive individuals were inpatients, and children aged less than 6 months have a high proportion (35.8%) in RSV infected individual [3]. Infants and children in specific subgroups, including premature infants and those with diseases such as chronic lung disease, congenital heart disease, interstitial lung disease, and cystic fibrosis, are at higher risk of RSV infection [4]. Furthermore, RSV infection is more likely to make an attack on adults with immune disorder, history of organ transplant, or age over 65 [5].

RSV infection is mainly histopathologically characterized by acute bronchiolitis, mucosal and submucosal edema, and luminal occlusion. At present, approaches for prevention of RSV infection include vaccination, antiviral drugs, intravenous immunoglobulin, monoclonal antibodies, and inhaled nanobodies [6, 7]. However, none of them has achieved perfect clinical outcomes due to a variety of factors such as high cost, adverse reaction, and doubtful efficacy. There is a need for efficacious antiviral treatments against RSV to be developed. N-acetyl-L-cysteine (NAC) is a thiol compound that can be directly used as a free radical scavenger and a precursor of reduced glutathione [8]. The antioxidant and anti-inflammatory actions of NAC in diverse respiratory diseases have been proved [9]. NAC is associated with alleviation in symptoms and delay in exacerbations of chronic obstructive pulmonary disease [10]. In addition, NAC has been found to inhibit COVID-19 replication and virus-induced apoptosis [11]. However, limited studies on the role of NAC in the prevention of RSV infection have been discussed. BEAS-2B is an immortalized but nontumorigenic epithelial cell line derived from human bronchial epithelial cells. It has been widely used as an in vitro cell model for enormous respiratory diseases studies including lung cancer due to its common recognition for medical science [12]. The present study was aimed at investigating the application of NAC in human bronchial epithelial cells BEAS-2B infected by RSV and focused on role of NAC on viral replication, antioxidant function, inflammatory response, and gene expression related to RSV.

## 2. Materials and Methods

### 2.1. Cell Harvest

Human laryngeal carcinoma cell line HEp-2 and transformed human bronchial epithelial cell line BEAS-2B (both purchased from ATCC, USA) were separately maintained in the DMEM equipped with penicillin (100 U/mL), streptomycin (100 mg/mL), and 10% fetal bovine serum (FBS) in an atmosphere with 5% CO_2_ saturated humidity at 37°C. The medium was refreshed every 2-3 days.

### 2.2. Preparation of Virus

The human A2 strain of RSV (ATCC) was propagated in HEp-2 cells and purified by sucrose cushion centrifugation at 65,000 × g for 2 h at 4°C. The cytopathic effect (CPE) caused by the virus was read after inoculation of 4 days and recorded as - indicating the absence of CPE, + indicating the presence of less than 25% cells, ++ indicating the presence of 25% to 50% cells, +++ indicating the presence of 50% to 75% cells, and ++++ indicating the presence of more than 75% cells. Virus titer was determined by 50% tissue culture infection dose (TCID50/mL). Virus stocks were stored at -80°C until used.

### 2.3. CCK-8 Assays

The cytotoxicity of NAC to BEAS-2B cells and the viability of RSV-infected BEAS-2B cells with or without NAC treatment were assessed by CCK-8 assays. For cytotoxicity assays, BEAS-2B cell suspension containing 1 × 10^7^ cells was placed in the 96-well plate and then treated with NAC at various concentrations (0, 0.1, 1, and 10 mM) for 24 h. The plate was added with serum-free medium supplemented with 10 *μ*L CCK-8 (5 mg/mL) in each well and reacted for 4 h, avoiding light. After aspiration of the supernatant, the cell pellet was maintained in 100 *μ*L DMSO. The absorbance was read at the 490 nm of wavelength.

### 2.4. RSV Infection and NAC Exposure

Once BEAS-2B cells reached 90% confluence, they were arranged into the control group, RSV group, NAC group, and RSV + NAC group. BEAS-2B cells in the control group were incubated in 3 mL DMEM (without penicillin-streptomycin solution and FBS) and UV-inactivated RSV confirmed by plaque assay for 24 h. BEAS-2B cells in the NAC group were incubated in 3 mL DMEM and UV-inactivated RSV, followed by exposure to 1 mM NAC [13] for 24 h. BEAS-2B cells in the RSV group were incubated in 500 *μ*L DMEM (without penicillin-streptomycin solution and FBS) and then infected by RSV at a multiplicity of infection (MOI) of 0.5 for 24 h [14]. BEAS-2B cells in the NAC group were exposed to 1 mM NAC for 1 h, infected by RSV at a MOI of 0.5, shaken every 30 min for 2 h, and incubated in 2.5 mL DMEM (without penicillin-streptomycin solution and FBS) for 24 h.

### 2.5. Plaque Assay

Once BEAS-2B cells reached 90% confluence, they were inoculated on 12-well plates and then infected with RSV at a MOI of 0.5 for 24 h. After rinsing in phosphate-buffered solution (PBS) to remove RSV, BEAS-2B cells were maintained in fresh medium containing 0.9% methylcellulose in an atmosphere with 5% CO_2_ saturated humidity at 37°C for 4-5 days for the formation of syncytia. Afterwards, BEAS-2B cells were fixed with 3% paraformaldehyde (Solarbio, China) for 30 min and stained with 1% crystal violet (Solarbio) for 30 min. The number of virus-induced plaques formed was counted under a light microscope.

### 2.6. Detection of Oxidative Stress Substances

BEAS-2B cells were incubated with 10 *μ*mol/L 2′, 7′-Dichlorodihydrofluoresce in diacetate (DCFH-DA) (Beyotime, Nantong, China) for 30 min, avoiding light, and detected by flow cytometry (FACS Calibur, BD FACSCalibur Becton Dickinson, USA) (the excitation wavelength: 488 nm; the emission wavelength: 525 nm) to examine reactive oxygen species (ROS) production. BEAS-2B cell suspension was collected after centrifugation (12000 × g, 10 min). The concentrations of superoxide dismutase (SOD) (A001-3-2, Nanjing Jiancheng Bioengineering Institute, Nanjing, China) and malondialdehyde (MDA) (A003-4-1, Nanjing Jiancheng Bioengineering Institute) and the contents of the nonenzymatic antioxidant system (GSH, GSSG, and GSH/GSSG ratio; Beyotime, Shanghai, China) were detected using commercial assay kits according to the operating instructions.

### 2.7. Enzyme-Linked Immunosorbent Assay (ELISA)

The production of TNF-*α*, IL-6, IL-1*β*, IL-18, and MUC5AC in the BEAS-2B cell supernatant were evaluated by the ELISA method using commercial assay kits (Sangon Biotech, Shanghai, China) according to the operating instructions.

### 2.8. Microarray Data Acquisition

The mRNA expression profile was obtained from GSE6802 deposited in the Gene Expression Omnibus (GEO). The GSE6802 was generated on the GPL571 platform, including three independent replicates for BEAS-2B cells stimulated with the bronchial pathogen RSV and UV-inactivated RSV. The Limma package loaded in the R/Bioconductor software was employed to analyze differentially expressed mRNAs, using |log2 (fold change [FC])| >2.5 and adjusted *p* < 0.05 as cutoff values, between BEAS-2B cells stimulated with RSV and UV-inactivated RSV.

### 2.9. Quantitative Real-Time PCR

The total RNA was isolated from BEAS-2B cells using TRIzol reagents (Invitrogen, USA) and then the PrimeScript RT kit (Takara, Dalian, China) was employed for cDNA synthesis. The SYBR-Green PCR kit (Roche Diagnostics, USA) was use for RT-qPCR through a StepOnePlus Real-Time PCR system (Applied Biosystems, USA). The primer sequence information were as follows: MUC5AC: 5′-TGATCATCCAGCAGCAGGGCT-3′ (sense) and 5′-GTGATGGCATGGACTGTGGT-3′ (antisense); HSPA6: 5′-ATGGTTCATGAAGCCGAGCA-3′ (sense) and 5′-TCTTGCATTTTGCGCCTGTC-3′ (antisense); RSV fusion (RSV-F): 5′-TGCAGTGCAGTTAGCAAAGG-3′ (sense) and 5′-TCTGGCTCGATTGTTTGTTG-3′ (antisense); TNF-*α*: 5′-ATGAGCACTGAAAGCATGATCCGG-3′ (sense) and 5′-GCAATGATCCCAAAGTAGACCTGCCC-3′ (antisense); IL-6: 5′-ATGAACTCCTTCTCCACAAGCGC-3′ (sense) and 5′-GAAGAGCCCTCAGGCTGGACTG-3′ (antisense); IL-1*β*: 5′-ATGGCAGAAGTACCTAAGCTCGC-3′ (sense) and 5′-ACACAAATTGCATGGTGAAGTCAGTT-3′ (antisense); IL-18: 5′-TCTACTGGTTCAGCAGCCATC-3′ (sense) and 5′-ACTGACCACTGAACTTGAAGGTA-3′ (antisense); GAPDH: 5′-ATGGAGAAGGCTGGGGCTC-3′ (sense) and 5′-AAGTTGTCATGGATGACCTTG-3′ (antisense). The results were analyzed using the 2-*ΔΔ*Ct method and normalized to GAPDH.

### 2.10. Immunoblotting Analysis

BEAS-2B cells were lysed with RIPA buffer, with the total protein extracted. The protein per lane was loaded onto a 10% SDS-PAGE and transferred to the PVDF membranes (GE Healthcare, USA). The membranes were probed with primary antibodies against EGFR (ab52894, Abcam, Cambridge, UK), p-EGFR (ab32430, Abcam), HSPA6 (ab212044, Abcam), and GAPDH (ab8245, Abcam) overnight at 4°C. Then, the membranes were rinsed three times and incubated with HRP-conjugated secondary antibodies for 1 h at room temperature, and HRP substrates were added to visualize the protein bands. The housekeeping gene GAPDH was used as a control for normalization. Densitometric analysis of immunoblots was performed using ImageJ software (USA).

### 2.11. Statistical Analysis

Data analysis was performed using the GraphPad prism software Version 8.0 (CA, USA). All data were presented as mean ± standard deviation and analyzed by unpaired *t* test between two groups and by one-way ANOVA method. If the possibility (*p*) of difference were less than 0.05, the difference was considered statistically significant.

## 3. Results

### 3.1. NAC Protected BEAS-2B Cell Survival against RSV Infection

**Figure 1:**
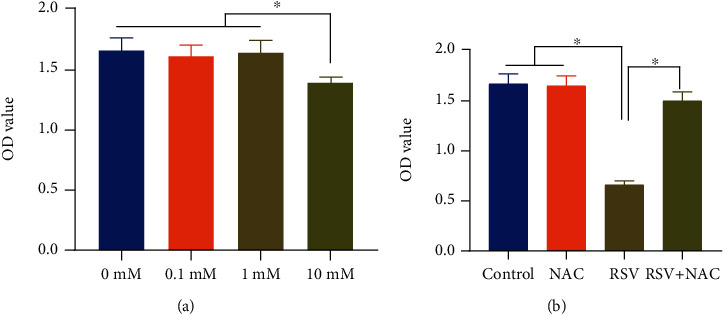
NAC treatment protects BEAS-2B cell survival against RSV infection. (a) BEAS-2B cells were exposed to NAC at 0, 0.1, 1, and 10 mM concentrations for 24 h, and CCK-8 assays were performed to assess the cytotoxicity of NAC to BEAS-2B cells. (b) CCK-8 assays were performed to examine in RSV-infected BEAS-2B cells with or without 1 mM NAC treatment. ∗ indicates *p* < 0.05.

To examine the cytotoxicity of NAC to BEAS-2B cells, we exposed BEAS-2B cells to NAC at 0, 0.1, 1, and 10 mM concentrations for 24 h, respectively. Results of CCK-8 assay found that NAC treatment at doses of 0, 0.1, and 1 mM did not affect BEAS-2B cell viability, while 10 mM significantly inhibited BEAS-2B cell viability (Figure 1(a)). Therefore, we exposed BEAS-2B cells to 1 mM NAC for further experiments. Next, we examined the effect of NAC on the survival of RSV-infected BEAS-2B cells, we exposed RSV-infected BEAS-2B cells to 1 mM NAC. Results of CCK-8 assay (Figure 1(b)) showed no significant difference regarding BEAS-2B cell survival between the control group and NAC group, suggesting no cytotoxic effect of 1 mM NAC on BEAS-2B cells. Compared with BEAS-2B cells in the control group and NAC group, BEAS-2B cells in the RSV group showed reduced cell survival. The cell survival was higher in the RSV + NAC group than in the RSV group, which indicated that NAC treatment could protect BEAS-2B cells against RSV infection.

### 3.2. Anti-inflammatory Properties of NAC in RSV-Infected BEAS-2B Cells

**Figure 2:**
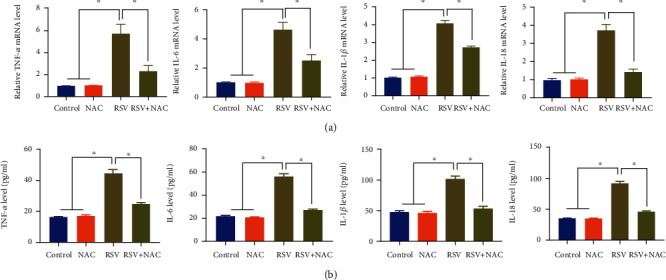
Anti-inflammatory properties of NAC in RSV-infected BEAS-2B cells. Quantitative real-time PCR (a) and ELISA (b) were performed to examine the production of inflammatory cytokines, TNF-*α*, IL-6, IL-1*β*, and IL-18, from BEAS-2B cells. ∗ indicates *p* < 0.01.

We next investigated the effect of NAC on the production of inflammatory cytokines from BEAS-2B cells. Results of quantitative real-time PCR (Figure 2(a)) found that the mRNA expressions of TNF-*α*, IL-6, IL-1*β*, and IL-18 were increased in the BEAS-2B cells in the RSV group in contrast to the control group and NAC group. Besides, the mRNA expressions of TNF-*α*, IL-6, IL-1*β*, and IL-18 were lower in the RSV + NAC group than in the RSV group. The control group and NAC group exhibited no significant difference with regard to the mRNA expressions of inflammatory cytokines. We also detected the concentrations of TNF-*α*, IL-6, IL-1*β*, and IL-18 in the cell supernatants from four groups of BEAS-2B cells using ELISA method. The ELISA results (Figure 2(b)) were consistent with quantitative real-time PCR results. RSV infection strikingly increased the release of TNF-*α*, IL-6, IL-1*β*, and IL-18 in the cell supernatants of BEAS-2B cells, and these increases were prevented by NAC treatment.

### 3.3. Antioxidant Properties of NAC in RSV-Infected BEAS-2B Cells

**Figure 3:**
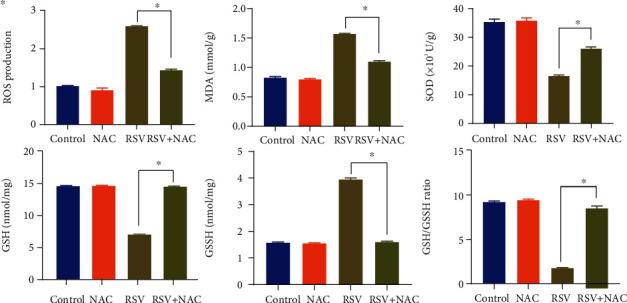
Antioxidant properties of NAC in RSV-infected BEAS-2B cells by detecting the ROS production, MDA level, SOD activity, GSH, GSSH, and GSH/GSSH ratio. ∗ indicates *p* < 0.01.

Oxidative stress-dependent events are associated with virus-infection mechanisms and the production of inflammatory mediators in human epithelial cells. To study the effect of NAC on oxidative stress during virus infection, we detected the ROS production, the MDA level, and the SOD activity and the GSH/GSSG ratio in RSV-infected BEAS-2B cells. We failed to observe significant differences on these levels of oxidative stress substances between the control group and NAC group. The ROS production and MDA level were increased, but the SOD activity and GSH/GSSG ratio were declined in BEAS-2B cells following RSV infection, whereas the NAC treatment weakened RSV-induced increase of ROS and MDA as well as decline of SOD and GSH/GSSG ratio in BEAS-2B cells (Figure 3). These data suggested that NAC exerted an antioxidant influence on BEAS-2B cells during RSV infection.

### 3.4. NAC Inhibited EGFR Activation and Mucin Synthesis in RSV-Infected BEAS-2B Cells

**Figure 4:**
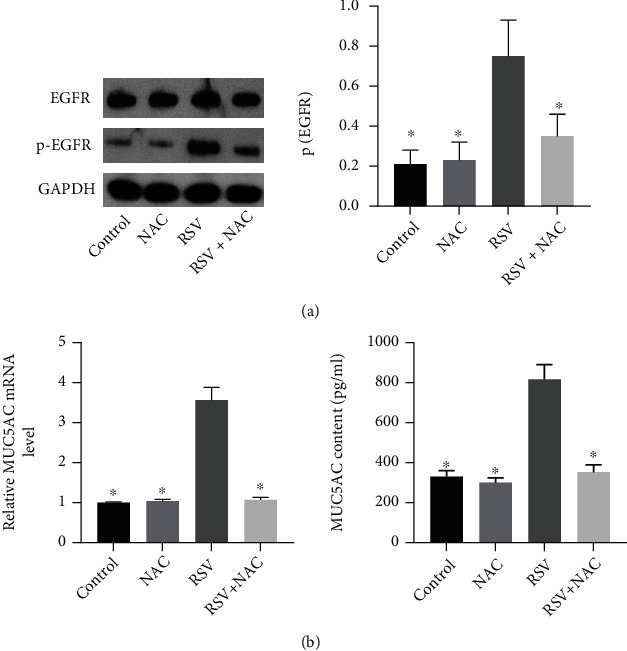
NAC inhibits EGFR activation and mucin synthesis in RSV-infected BEAS-2B cells. (a), Immunoblotting analysis of EGFR and p-EGFR in RSV-infected BEAS-2B cells. (b), Quantitative real-time PCR and ELISA detection of MUC5AC mRNA expression and MUC5AC contents. ∗ indicates *p* < 0.05 compared with RSV group.

RSV induces airway epithelial inflammation by activation of EGFR, a tyrosine kinase receptor. Accordingly, we attempt to study the effects of NAC on EGFR in RSV-infected BEAS-2B cells. Basal level of EGFR phosphorylation in RSV-infected BEAS-2B cells was higher than that in control BEAS-2B cells without outside stimuli and with NAC only. NAC treatment was shown to reduce the level of EGFR phosphorylation in RSV-infected BEAS-2B cells (Figure 4(a)). Either at baseline or after RSV infection, there is no significant difference of total EGFR between NAC-treated BEAS-2B cells and control BEAS-2B cells. MUC5AC is a well-known mucus protein that is produced by airway surface epithelium and involved in the development of asthma. We therefore investigated the effects of NAC on MUC5AC expression in RSV-infected BEAS-2B cells. It was found that RSV infection elevated the mRNA level of MUC5AC in BEAS-2B cells and the production of MUC5AC from the BEAS-2B cell supernatant, which was reversed following NAC treatment (Figure 4(b)).

### 3.5. NAC Inhibited HSPA6 Expression in RSV-Infected BEAS-2B Cells

**Figure 5:**
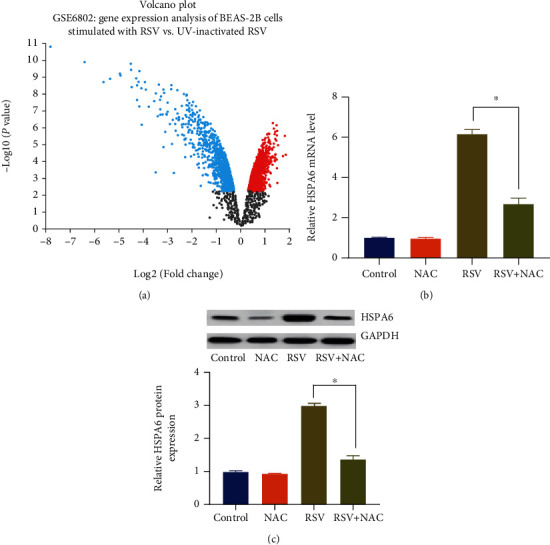
NAC treatment inhibits HSPA6 expression in RSV-infected BEAS-2B cells. (a), The volcano plot showing differentially expressed genes between BEAS-2B cells stimulated with UV-inactivated RSV and BEAS-2B cells stimulated with the bronchial pathogen RSV. (b), Quantitative real-time PCR was performed to determine the mRNA expression of HSPA6 in RSV-infected BEAS-2B cells with or without NAC treatment. (c) Immunoblots of HSPA6 protein and their densitometric analysis in RSV-infected BEAS-2B cells with or without NAC treatment. ∗ indicates *p* < 0.01.

We differentially analyzed the raw data of gene expression profile of GC patients in the GSE6802 and identified differentially expressed genes between BEAS-2B cells stimulated with the bronchial pathogen RSV and BEAS-2B cells stimulated with UV-inactivated RSV (Figure 5(a)). We used |log2 (FC)| >2.5 and adjusted *p* < 0.05 as cutoff values and top 20 differentially expressed genes between BEAS-2B cells stimulated with UV-inactivated RSV and RSV-infected BEAS-2B cells (Table 1). The gene encoding heat shock protein family A (Hsp70) member 6 (HSPA6) exhibited the largest fold change. In order to explore the mechanism behind RSV infection and NAC protection, we determined the expression of HSPA6 expression in RSV-infected BEAS-2B cells with or without NAC administration. Results of quantitative real-time PCR (Figure 5(b)) and immunoblotting analysis (Figure 4(c)) showed that the RSV group exhibited elevated mRNA and protein expressions of HSPA6 compared with the control group and NAC group, whereas the RSV group showed declined mRNA and protein expressions of HSPA6 compared with the RSV + NAC group. These data revealed that RSV infection led to an increased HSPA6 expression and NAC inhibited RSV-induced HSPA6 expression in BEAS-2B cells.

### 3.6. Antiviral Activity of NAC in RSV-Infected BEAS-2B Cells

**Figure 6:**
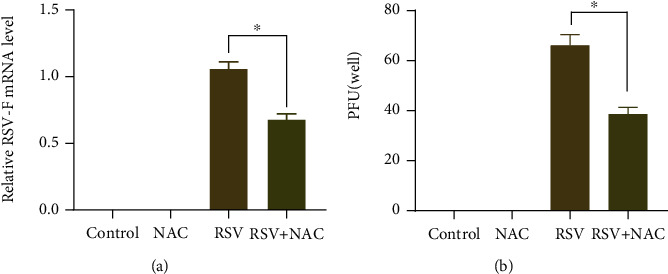
Antiviral activity of NAC in RSV-infected BEAS-2B cells. Quantitative real-time PCR (a) and plaque assay (b) were performed to examine the mRNA expression of RSV-F and the number of virus-induced plaques formed in RSV-infected BEAS-2B cells. ∗ indicates *p* < 0.01.

The antiviral effect of NAC on RSV-infected BEAS-2B cells was explored in this part. As determined by quantitative real-time PCR, the expression of RSV-F was increased in BEAS-2B cells following RSV infection, and NAC administration negated RSV-induced increase of RSV-F expression (Figure 6(a)). A plaque assay revealed that the number of virus-induced plaques formed was increased in BEAS-2B cells following RSV infection, whereas the number of virus-induced plaques was reduced in RSV-infected BEAS-2B cells after exposure to 1 mM NAC (Figure 6(b)).

## 4. Discussion

RSV infection is one of the main causes of infant hospitalization and death. Although antiviral drugs and monoclonal antibodies are the two main approaches against RSV infection, treatment is supportive. However, clinical and economic burden of RSV infection are increasing worldwide due to high cost of drugs, the lack of specificity of ribavirin, and application of palivizumab in specific populations. It is in urgent need of development of new antiviral medicines to prevent RSV infection. In this study, the effects of NAC on EGFR activation, mucin synthesis, inflammatory response, oxidative stress, and viral replication were investigated in BEAS-2B cells.

Initially, we found NAC protected BEAS-2B cell survival against RSV infection. In the past decade, a comprehensive understanding of RSV host pathogen interaction and virus replication has provided the basis for antiviral treatment of RSV-induced diseases [15]. RSV-F is a fusion protein which expresses on the virion surface and mediates fusion of virus membrane and host cell membrane [16]. In order to further confirm antiviral activity of NAC in RSV, we performed quantitative real-time PCR and observed that elevated expression of RSV-F was revealed in RSV infected-BEAS-2B cells; besides, RSV-F expression was decreased after NAC administration. These results were supported by the plaque assay in our study. Antiviral activity of NAC has been proved in previous studies. NAC contributed to alleviate Coxsackievirus B type 3-induced myocardial injury, suppress viral replication, and inhibit inflammatory response [17].

RSV virus activity and load are related to the severity of the disease and also determine the degree of inflammation [18]. The anti-inflammatory property of NAC was confirmed in our study. In contrast with other 3 groups, data from quantitative real-time PCR showed the RSV group had the highest level of TNF-*α*, IL-6, IL-1*β*, and IL-18 mRNA expression in BEAS-2B cells. In addition, these expressions were lower in the RSV + NAC group than that in the RSV group. The ELISA results in this study also supported the findings above. The present study also found that BEAS-2B cells following RSV infection showed increased ROS production and MDA level but declined SOD activity and GSH/GSSG ratio. However, this trend was weakened in the infected BEAS-2B cells treated with NAC. Glutathione is a tripeptide observed in all cells except erythrocytes, and it plays an important role as free radical scavenger, inhibitor of lipid peroxidation, and in detoxification [19]. The ratio of reduced/oxidized glutathione (GSH/GSSG) is an indicator of cellular health, and this ratio is declined in tissue or cells under oxidative stress [20]. NAC is the acetylated form of the amino acid L-cysteine and a precursor to GSH and has been known for a long time as a powerful antioxidant [21]. NAC has been demonstrated to reverse the reduction in the GSH/GSSG ratio in the setting of oxidative stress [22]. The inhibitory effects of NAC on EGFR activation and mucin synthesis in RSV-infected BEAS-2B cells were also proved in our study. RSV triggers airway epithelial inflammation by activation of the EGFR, and the inhibition of EGFR could reduce RSV infection by promoting endogenous epithelial antiviral defenses [23]. NAC was previously found to restore the sensitivity of gefitinib-resistant lung cancer cells to gefitinib that is a major epithelial growth factor receptor tyrosine kinase inhibitor, suggesting the inhibitory effects of NAC on EGFR activation in epithelial cells [24]. With regard to NAC inhibition on mucin hypersecretion caused by RSV infection [25], a previous study, similar as our study, reported that NAC reduce airway inflammation and responsiveness, goblet cell hyperplasia, and lung fibrosis by increasing levels of ROS, nuclear factor erythroid 2-related factor 2, and MUC5AC protein [26]. The present study analyzed the HSPA6 expression in RSV-infected BEAS-2B cells treated with and without NAC. We discovered NAC treatment reduced mRNA and protein expressions of HSPA6. HSPA6 is a strictly stress-inducible member of Hsp70 family and has low or nondetectable expression levels in most cells [27]. However, it has been found to be expressed in response to cellular stresses including viral infection, such as tick-borne encephalitis virus [28] and Enterovirus 71 [29]. HSPA6 was also shown to be expressed at high levels in neurodegenerative diseases and in tissue or cells under oxidative stress [30]. Viruses could regulate host HSPs at different levels such as transcription, translation, posttranslational modification, and cellular localization. In response to physiological stresses including viral infection and oxidative stress, heat shock factors are produced from chaperone and binds to heat-shock elements in the Hsp gene promoters. NAC could reduce Hsp70 expression levels in HepG2 cells treated by acrylamide, a harmful chemical affecting the liver [31], which is partially similar with our study.

In summary, NAC treatment made a positive impact on culture of BEAS-2B cells infected by RSV. The antiviral properties, anti-inflammatory action, and antioxidant effect of NAC in human bronchial epithelial cells with RSV infection were well established in this study. Further analysis of the antiviral effect of NAC regulation of HSPA6 through genetic or epigenetic mechanism should be discussed to better know the mechanism of NAC in RSV infected diseases.

## Figures and Tables

**Table 1:** There were top 20 differentially expressed genes (|log2 (FC)| >2.5 and adjusted *p* < 0.05) between BEAS-2B cells stimulated with UV-inactivated RSV and RSV-infected BEAS-2B cells.

Adj. *p* val	*t*	*B*	logFC	Gene symbol	Full name
6.24E-08	-71.04	15.97	-7.94	HSPA6	Heat shock protein family A (Hsp70) member 6
2.64E-07	-51.32	14.87	-4.60	ATF3	Activating transcription factor 3
4.64E-07	-45.76	14.37	-4.59	CXCL8	C-X-C motif chemokine ligand 8
5.62E-07	-42.62	14.04	-5.05	FOSB	FosB proto-oncogene, AP-1 transcription factor subunit
8.14E-07	-38.92	13.58	-4.37	NR4A1	Nuclear receptor subfamily 4 group A member 1
8.14E-07	-38.57	13.53	-5.46	NR4A3	Nuclear receptor subfamily 4 group A member 3
1.00E-06	-36.37	13.21	-4.28	SIK1	Salt inducible kinase 1
1.28E-06	-34.54	12.92	-3.43	GEM	GTP binding protein overexpressed in skeletal muscle
1.28E-06	-34.18	12.86	-2.91	DNAJB1	DnaJ heat shock protein family (Hsp40) member B1
1.56E-06	-32.94	12.64	-4.56	FOS	Fos proto-oncogene, AP-1 transcription factor subunit
1.76E-06	-32.19	12.51	-4.16	CXCL1	C-X-C motif chemokine ligand 1
1.82E-06	-31.63	12.40	-2.92	HSPA1B	Heat shock protein family A (Hsp70) member 1B
1.82E-06	-31.42	12.36	-3.24	NEDD9	Neural precursor cell expressed, developmentally downregulated 9
1.89E-06	-31.03	12.28	-2.66	MAFF	MAF bZIP transcription factor F
2.69E-06	-29.48	11.97	-3.21	TNFAIP3	TNF alpha-induced protein 3
2.77E-06	-29.20	11.91	-3.56	SNAI1	Snail family transcriptional repressor 1
3.72E-06	-27.54	11.53	-3.34	IL6	Interleukin 6
5.85E-06	-25.74	11.08	-4.34	NR4A2	Nuclear receptor subfamily 4 group A member 2
6.50E-06	-25.18	10.93	-3.62	HEY1	Hes-related family bHLH transcription factor with YRPW motif 1
7.64E-06	-24.45	10.73	-3.06	JUNB	JunB proto-oncogene, AP-1 transcription factor subunit

## Data Availability

The data used to support the findings of this study are included within the article.
